# Supplementary material for: Correlation between Structural and Functional Changes in Patients with Raised Intraocular Pressure Due to Graves’ Orbitopathy
Source: Diagnostics (Basel). 2024 Mar 20;14(6):649. doi: 10.3390/diagnostics14060649 (PMC10968800; doi:10.3390/diagnostics14060649)
Supplement: Supplementary file 1 [file diagnostics-14-00649-s001.zip › diagnostics-2876312-supplementary.pdf]

Supplementary Materials:

Table S1 *Post-hoc* analysis of differences in the examined clinical parameters: Mann-Whitney U test. GO - Graves' orbitopathy, GO IOP - Graves' orbitopathy with elevated intraocular pressure, POAG- primary open-angle glaucoma; IOP- intraocular pressure; CCT- central corneal thickness; BCVA - best corrected visual acuity

Post-hoc comparisons	P value		
	GO vs GO IOP	GO vs POAG	GO IOP vs POAG
IOP (mmHg)	<0.001	<0.001	0.001
CCT (μm)	0.018	<0.001	0.086
Exophthalmometry (mm)	0.002	<0.001	<0.001
BCVA	0.007	<0.001	0.005

Table S2: Differences in the classification of disease severity between investigated groups (EUGOGO Severity Classification); GO - Graves' orbitopathy, GO IOP - Graves' orbitopathy with elevated intraocular pressure.

		Group				P
		GO		GO IOP		
		N	%	N	%	
Disease severity	Mild	83	88.3%	62	63.9%	<0.001
	Moderate	11	11.7%	35	36.1%	

Table S3: Duration of thyroid disease and orbitopathy: Kruskal-Wallis test; GO - Graves' orbitopathy, GO IOP - Graves' orbitopathy with elevated intraocular pressure, POAG- primary open-angle glaucoma.

Group		N	Min	Max	Centile			P
					25.	Median	75.	
Duration of thyroid disease (yrs)	GO	48	0.50	38.00	2.00	4.00	10.00	0.431
	GO IOP	50	0.50	30.00	2.88	5.00	13.00	
Duration of orbitopathy (yrs)	GO	48	0.50	13.00	1.00	2.00	3.00	0.435
	GO IOP	50	0.00	19.00	1.00	2.00	4.00	

Table S4. *Post-hoc* testing for differences in clinical changes with respect to optic nerve parameters: Mann-Whitney U test. GO - Graves' orbitopathy, GO IOP - Graves' orbitopathy with elevated intraocular pressure, POAG- primary open-angle, RNFL- retinal nerve fibre layer, c/d - cup/disc

Post-hoc comparisons	P value		
	GO vs GO IOP	GO vs POAG	GO IOP vs POAG
RNFL (μm)	0.907	<0.001	<0.001
Rim area (mm^2)	0.001	<0.001	<0.001
Disc area (mm^2)	0.145	0.218	0.989
Average c/d ratio	<0.001	<0.001	<0.001
Vertical c/d ratio	<0.001	<0.001	<0.001
Cup volume (mm^3)	0.007	<0.001	<0.001

Table S5: Differences in selected risk factors presented through categorical variables between participants in the GO and GO IOP groups. GO - Graves' orbitopathy, GO IOP - Graves' orbitopathy with elevated intraocular pressure, COPD - chronic obstructive pulmonary disease.

		Group				
		GO		GO IOP		P
		N	%	N	%	
Smoking	Yes	**27**	56.3%	27	54.0%	0.823
	No	21	43.8%	23	46.0%	
Stress at the time of diagnosis of thyroid disease	Yes	26	54.2%	15	30.0%	0.015
	No	22	45.8%	35	70.0%	
Diabetes	Yes	5	10.4%	4	8.0%	0.679
	No	43	89.6%	46	92.0%	
Arterial hypertension	Yes	11	22.9%	15	30.0%	0.427
	No	37	77.1%	35	70,0%	
Hyperlipidemia	Yes	10	20.8%	6	12.0%	0.237
	No	38	79.2%	44	88.0%	
Depression	Yes	4	8.3%	4	8.0%	0.952
	No	44	91.7%	46	92.0%	
Asthma/ Chronic obstructive pulmonary disease	Yes	4	8.3%	1	2.0%	0.154
	No	44	91.7%	49	98.0%	

Table S6: Differences in family history between participants in the GO and GO IOP groups.

		Group				
		GO		GO IOP		P
		N	%	N	%	
Family history	Negative	27	56.3%	28	56.0%	0.706
	Glaucoma	2	4.2%	5	10.0%	
	Thyroid disease	16	33.3%	14	28.0%	
	Glaucoma + thyroid disease	3	6.3%	3	6.0%	
